# Perirenal fat thickness contributes to the estimated 10-year risk of cardiovascular disease and atherosclerotic cardiovascular disease in type 2 diabetes mellitus

**DOI:** 10.3389/fendo.2024.1434333

**Published:** 2024-07-08

**Authors:** Wei Wang, Feng Yan Lv, Mei Tu, Xiu Li Guo

**Affiliations:** National Metabolic Management Center, Longyan First Affiliated Hospital of Fujian Medical University, Longyan, Fujian, China

**Keywords:** perirenal adipose tissue, perirenal fat thickness, Framingham risk scores, China-PAR equation risk scores, cardiovascular disease risk, type 2 diabetes mellitus

## Abstract

**Objective:**

Perirenal adipose tissue (PAT) has emerged as a potential therapeutic target for cardiovascular disease (CVD). However, the relationship between increased perirenal fat thickness (PrFT) and CVD risks in individuals with type 2 diabetes mellitus (T2DM) remains uncertain. This study aimed to evaluate the association between PrFT and the estimated 10-year risk of CVD and atherosclerotic cardiovascular disease (ASCVD) in T2DM.

**Method:**

The final analysis included 704 participants. PrFT was quantified using non-enhanced computed tomography scans, while the estimated 10-year CVD and ASCVD risk assessments were based on the Framingham and China-PAR equation risk scores, respectively. Multiple regression analysis was employed to analyze the correlation between PrFT and these risk scores.

**Results:**

Higher quartiles of PrFT displayed elevated Framingham and China-PAR equation risk scores (*P*<0.001). After adjusting for cardiometabolic risk factors and visceral fat area, PrFT remained significantly correlated with Framingham equation risk scores in men (*β*=0.098, *P*=0.036) and women (*β*=0.099, *P*=0.032). Similar correlations were observed between PrFT and China-PAR equation risk scores in men (*β*=0.106, *P*=0.009) and women (*β*=0.108, *P*=0.007). Moreover, PrFT emerged as an independent variable associated with a high estimated 10-year risk of CVD and ASCVD, with odds ratios (ORs) of 1.14 (95% CI: 1.04-1.25, *P*=0.016) in men and 1.20 (95% CI: 1.11-1.31, *P*<0.001) in women for high estimated CVD risk, and ORs of 1.22 (95% CI: 1.08-1.41, *P*=0.009) in men and 1.34 (95% CI: 1.12-1.60, *P*<0.001) in women for high estimated 10-year ASCVD risk. Furthermore, restricted cubic spline analyses confirmed a nonlinear relationship between PrFT and high estimated CVD and ASCVD risk in both genders (*P* for nonlinearity and overall < 0.05).

**Conclusions:**

PrFT contributed as an independent variable to the estimated 10-year risk of CVD and ASCVD in T2DM.

## Introduction

Type 2 diabetes mellitus (T2DM) represents a metabolic disorder characterized by chronic hyperglycemia, posing a significant global health threat due to its escalating prevalence (1). It is intricately intertwined with cardiovascular disease (CVD), leading to a twofold increase in the risk of all-cause mortality, with CVD emerging as the primary cause of death, as demonstrated by an extensive prospective study involving 512,869 adults in China (2). Concurrently, T2DM often manifests alongside obesity (3), dyslipidemia (4), and insulin resistance (5), which were recognized cardio-metabolic risk factors exacerbating susceptibility to CVD. Despite strides in glycemic management, the incidence and mortality of CVD persist, particularly among T2DM cohorts. Consequently, the latest guidelines advocate a paradigm shift in CVD management within T2DM towards a personalized, patient-centered approach, accentuating early intervention of risk factors based on intensive glycemic control to mitigate CVD risk (6). The escalating prevalence of obesity contributes significantly to the burgeoning incidence of T2DM. Meanwhile, epidemiological investigations have underscored obesity as an independent risk factor for CVD, even after adjusting for other cardiometabolic risk factors (7). Dysfunctional adipose tissue accumulation, particularly in visceral depots, plays a crucial role in T2DM and CVD pathogenesis. Recent advancements in obesity and CVD risk underscore the significant heterogeneity in body fat distribution, emphasizing the role of visceral adiposity as a critical depot linked to increased risk of both CVD and T2DM (8). The classification of visceral adipose tissue (VAT) is intricate, and discerning which specific VAT correlates with CVD risk is essential for effective risk assessment.

Perirenal adipose tissue, a specific type of VAT situated in the retroperitoneal space, has recently attracted clinical insights owing to its special roles in the metabolism and cardiovascular system, speculated as a potential treatment target for CVD (9, 10). Current protocols for managing T2DM recommend annual evaluation of CVD risk factors and the utilization of validated risk prediction models, such as the Framingham (11)and China-PAR equation risk scores (12), to estimate the 10-year risk of CVD or atherosclerotic cardiovascular disease (ASCVD). Perirenal fat thickness (PrFT) emerges as a validated index proficient in accurately representing the PAT mass (13). Previous studies have demonstrated a close association between increased PAT and cardiometabolic risk factors, such as hypertension (14), metabolic dysfunction-associated fatty liver disease (15), and increased carotid intima-media thickness (16). However, limited data has investigated the association between increased PAT and estimated CVD or ASCVD risk. Therefore, this study aimed to evaluate the correlation of PrFT with the estimated 10-year risk of CVD or ASCVD risk in T2DM.

## Materials and methods

### Participants and study design

**Figure 1:**
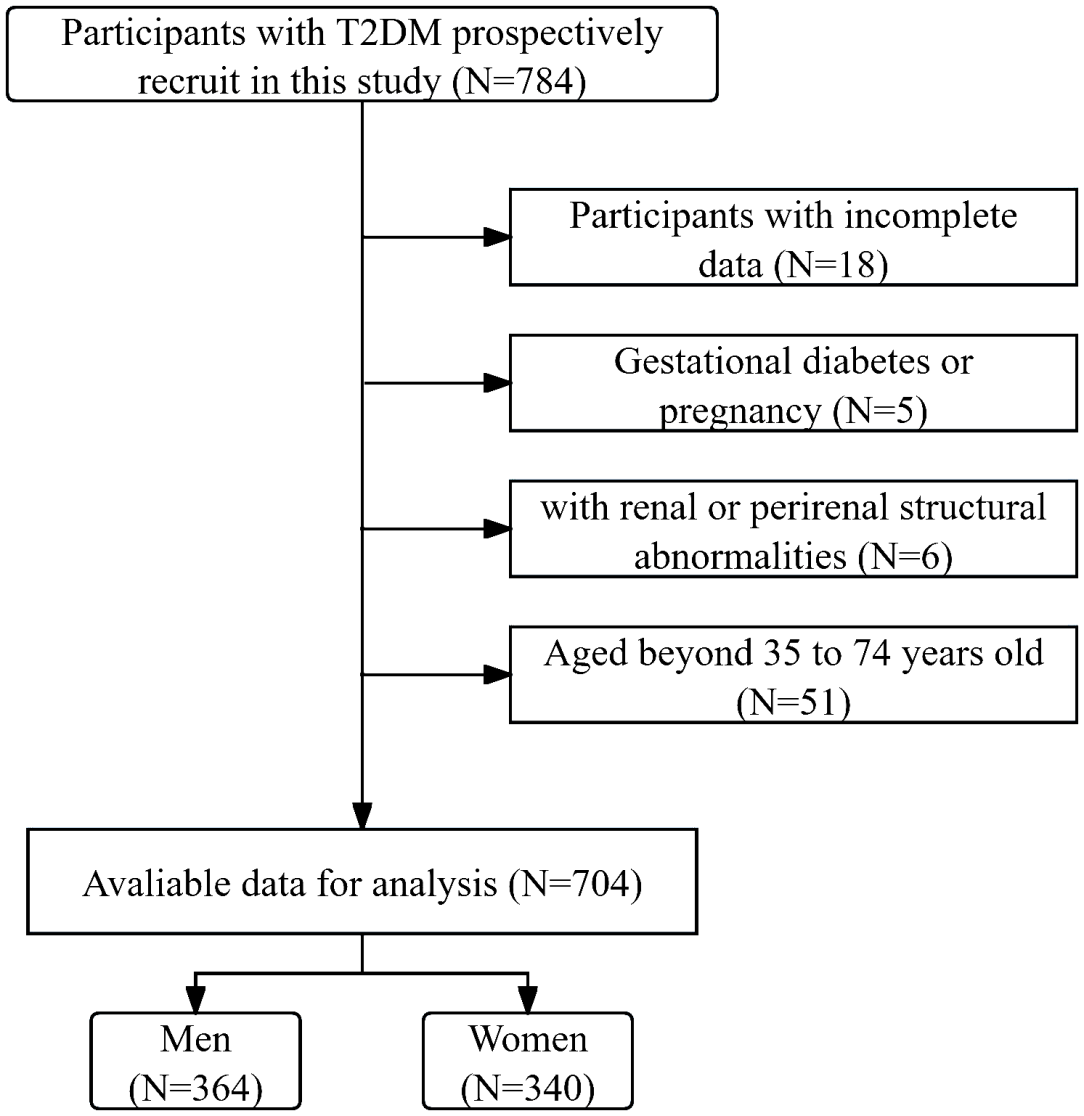
The flow diagram illustrates the enrollment process throughout the study.

In this cross-sectional study, participants with T2DM admitted to the National Metabolic Management Center at Longyan First Affiliated Hospital of Fujian Medical University were consecutively recruited from January 2023 to March 2024. Written informed consent was obtained from all participants under the ethical guidelines established by the Ethical Committee of our hospital (IC-2020-069), adhering rigorously to the principles outlined in the Declaration of Helsinki throughout the study. Participants were excluded during the participant selection process if they met one of the following conditions: 1. aged beyond 35 to 74 years old, as Framingham risk scores were only applicable within this age range. 2. exhibited incomplete data. 3. experienced acute major cardiovascular events. 4. had renal abnormalities, such as renal or perirenal neoplasms, cysts, or a history of renal region surgery. 5. Gestational diabetes or pregnancy. After the participant selection period, 704 participants were included in the final analysis. **Figure 1** delineates the enrollment process in a flow diagram.

### Data collection and laboratory assessments

Clinical data were systematically collected by trained interviewers utilizing a standardized questionnaire and a comprehensive review of medical records and laboratory findings. Parameters encompassed demographic details such as age, gender, pertinent medical history, prior surgeries potentially affecting the renal structure, and family history of ASCVD, alongside current or previous medication usage. Additionally, diabetic duration, residential status, and habits regarding smoking and alcohol consumption were documented. Anthropometric measurements were conducted by skilled research nurses employing established protocols, encompassing waist circumference (WC), systolic blood pressure (SBP), and diastolic blood pressure (DBP).

Laboratory assessments were conducted utilizing fasting venous blood samples obtained between 8 and 9 am following an overnight fast, stored in standardized tubes containing dipotassium ethylenedinitrilo tetra-acetic acid. Serum levels of various parameters were determined employing standard methodologies. These included fasting blood glucose (FBG), serum insulin, glycated hemoglobin (HbA1c), as well as diabetic autoimmune antibodies (GADA, IAA, and ICA) to exclude type 1 diabetes. Additionally, measurements encompass creatinine, triglycerides (TGs), total cholesterol, high-density lipoprotein cholesterol (HDL-c), and low-density lipoprotein cholesterol (LDL-c). HbA1c was quantified using high-performance liquid chromatography with a D10 set (Bio-RAD), while biochemical indices were assessed via an auto-biochemical analyzer (Roche Diagnostics Corporation). Insulin sensitivity was evaluated using the homeostasis model assessment of insulin resistance (HOMA-IR), calculated as fasting serum insulin (µU/ml) multiplied by FBG (mmol/l) divided by 22.5 (17). These clinical and laboratory data will be stored in the National Metabolic Management Center’s unique information collection system for final analysis.

### Measurement ofperirenal fat thickness and visceral fat area

The measurement of PrFT was conducted following the method initially proposed by the Mayo Clinic in 2014 (18). Participants underwent unenhanced abdominal computed tomography scans covering the area from the pubic symphysis to the 10th thoracic vertebra to acquire renal and perirenal structural images. Two seasoned radiologists engaged in PrFT measurement, adhering to standardized protocols. Initially, PAT was delineated from adjacent tissues based on density criteria (window center: -100 HU, widths: -50 to -200 HU). Subsequently, PAT was defined as the distance from the renal tissue to the nearest visceral or muscular structure. Lastly, PrFT was quantified as the average maximum distance from the posterior aspect of the kidney to the inner edge of the abdominal wall along the plane encompassing the left and right renal veins.

The visceral fat area (VFA) measurement was conducted by skilled operators using a dual bioelectrical impedance analyzer (DUALSCANHDS-2000, Omron Healthcare Company, Japan), adhering to standardized protocols.

### Assessments of estimated 10-year CVD and ASCVD risk

Estimated 10-year CVD and ASCVD risk assessments were conducted using the Framingham and China-PAR equation risk scores, respectively. These scores are endorsed for predicting 10-year CVD and ASCVD risk by Chinese guidelines for DM management and were calculated according to previously published algorithms (11, 12). Framingham risk scores comprise variables including gender (male or female), age (years), TC levels (mmol/L), HDL levels (mmol/L), BP, smoking status (yes or no), and diabetes status (yes or no). According to the Framingham Risk Score (FRS), participants are stratified into high-risk (≥20%), intermediate-risk (10-20%), and low-risk (<10%) categories. The China-PAR equation incorporates variables such as gender (male or female), age (years), untreated SDP (mmHg), TC levels (mg/dl), HDL levels (mg/dl), WC, smoking status (yes or no), diabetes status (yes or no), geographic region (northern or southern China), urbanization status (urban or rural), and family history of ASCVD (yes or no). Participants are categorized based on the China-PAR equation risk score into high-risk (≥10%), intermediate-risk (5-10%), and low-risk (<5%) groups.

### Statistical analysis

The clinical and laboratory assessment data will eventually be exported in Excel form from our information storage system. The SPSS 26.0 software (IBM SPSS Inc.) was employed for statistical analysis. Participants were divided into four groups based on the quartiles of PrFT: Q1(<8.3mm), Q2 (8.3-12.9mm), Q3 (13.0-16.4mm), and Q4 (>16.4mm). Descriptive statistics are presented as means ± standard deviation (SD), and statistical differences among the PrFT quartiles were assessed using a one-way analysis of variance. Discrete variables are summarized in frequency tables (N, %), and the chi-squared (χ2) test or Fisher’s exact test was employed for comparing categorical variables. The association between PrFT and China-PAR or Framingham risk scores was assessed via Spearman correlation analysis, further elucidated by multiple regression analysis after adjusting the potential confounders reported in previous studies in three models. Model 1: adjustment for the diabetic duration, HbA1c, HOMA-IR, TG, and LDL-c. Model 2: additional adjustment for components of the Framingham and China-PAR risk prediction models like age, WC, TC, HDL-c, SBP, DBP, family ASCVD history, smoking, and urbanization. Model 3: further adjustment for the VFA and usage of glucagon-like peptide 1 receptor agonists (GLP-1 RAs) or sodium-glucose co-transporters type 2 inhibitors (SGL-T2is). Binomial logistic regression analysis was utilized to identify the independent variables of PrFT for high estimated 10-year CVD and ASCVD risk in three different models. Furthermore, the restricted cubic splines (RCS) analyses were conducted to evaluate the non-linear association between PrFT and high estimated 10-year CVD and ASCVD risk after full adjustment for Model 3. Statistical significance was set at *P*<0.05 (two-tailed).

## Results

### Baseline characteristics of participants

In total, 704 individuals with T2DM and complete data were included in the final analysis. The gender distribution was balanced, with 364 (51.7%) male participants. The mean age of the cohort was 53.5 ± 8.0 years, and the mean duration of diabetes was 8.3 ± 3.1 years. The mean Framingham and China-PAR equation risk scores were 18.7% ± 10.8% and 7.0% ± 5.4%, respectively. Additionally, 319 (45.3%) and 221 (31.4%) participants were categorized as high-risk for CVD or ASCVD according to Framingham and China-PAR equation risk scores, respectively.

### Clinical and laboratory characteristics of participants across PrFT quartiles

The clinical and laboratory characteristics of the participants, stratified by quartiles of PrFT, are summarized in **Table 1**. No significant differences were observed in age, HbA1c, creatinine levels, or the proportion of smokers and urban dwellers across the PrFT quartiles (*P*>0.05). However, significant differences were noted in components of the Framingham and China-PAR risk prediction models, including WC, TC, HDL-c, SBP, and DBP across the PrFT quartiles (*P*<0.05). Moreover, there were increasing trends in the Framingham and China-PAR equation risk scores with higher PrFT quartiles. Participants in the higher PrFT quartiles exhibited a greater prevalence of high-risk CVD and ASCVD, as well as hypertension (*P*<0.05).

**Table 1:** Clinical and laboratory characteristics of the study population based on the quartiles of PrFT(mm).

Variable	Total	Q1(<8.3)	Q2(8.3-12.9)	Q3(13.0-16.4)	Q4(>16.4)	*P*
Age (year)	54.3 ± 8.1	54.1 ± 8.8	53.1 ± 6.1	54.1 ± 7.1	55.8 ± 7.1	0.463
Diabetes duration (year)	8.3 ± 3.1	8.0 ± 4.2	8.6 ± 3.8	8.3 ± 2.9	8.4 ± 2.6	0.816
Men, n (%)	364(51.7)	108(61.4)	77(43.8)	92(50.0)	87(51.8)	0.010
WC (cm)	85.4 ± 7.1	80.2 ± 4.8	84.7 ± 6.0	87.0 ± 5.6	87.0 ± 7.8	<0.001
HbA1c (%)	8.4 ± 1.0	8.3 ± 0.8	8.4 ± 0.9	8.6 ± 1.1	8.3 ± 1.2	0.786
TG (mmol/L)	2.14 ± 1.39	1.14 ± 0.76	1.96 ± 1.17	2.25 ± 0.86	3.25 ± 1.69	<0.001
TC (mmol/L)	5.38 ± 1.21	5.07 ± 1.15	5.24 ± 1.21	5.64 ± 1.21	5.54 ± 1.21	<0.001
HDL-c (mmol/L)	1.09 ± 0.25	1.27 ± 0.28	1.11 ± 0.21	1.02 ± 0.18	0.94 ± 0.17	<0.001
LDL-c (mmol/L)	3.54 ± 0.95	3.24 ± 0.94	3.52 ± 0.91	3.83 ± 0.96	3.55 ± 0.95	<0.001
SBP (mmHg)	134.5 ± 16.5	118.5 ± 13.5	132.1 ± 12.3	140.5 ± 11.3	147.1 ± 13.7	<0.001
DBP (mmHg)	81.0 ± 8.8	74.3 ± 6.6	80.4 ± 7.3	83.8 ± 7.7	85.7 ± 8.9	<0.001
HOMA-IR	11.2 ± 6.2	6.6 ± 3.8	10.7 ± 5.6	13.0 ± 4.3	14.8 ± 7.2	<0.001
Hypertension, n (%)	266(37.8)	24(13.6)	43(24.4)	91(49.5)	108(64.3)	<0.001
Smoking, n (%)	193(27.4)	59(33.5)	43(24.9)	44(23.9)	47(28.0)	0.171
Urban, n (%)	368(52.3)	90(51.1)	95(54.0)	92(50.0)	91(51.1)	0.892
Family ASCVD history, n (%)	22(3.1)	6(3.4)	5(2.8)	5(2.7)	6(3.6)	0.959
Framingham risk score	18.7 ± 10.8	10.1 ± 6.1	14.6 ± 7.1	21.5 ± 8.7	28.9 ± 10.4	<0.001
Low-risk, n (%)	186(26.4)	122(69.3)	45(25.6)	16(8.7)	3(1.8)	<0.001
Intermediate-risk, n (%)	199(28.3)	32(18.2)	101(57.4)	51(27.7)	15(8.9)	<0.001
High-risk, n (%)	319(45.3)	22(12.5)	30(17.0)	117(63.6)	150(89.3)	<0.001
China-PAR equation risk score	7.0 ± 5.4	3.0 ± 2.3	4.5 ± 2.8	7.7 ± 4.5	13.0 ± 5.1	<0.001
Low-risk, n (%)	343(48.7)	149(84.7)	129(73.3)	63(34.2)	2(1.2)	<0.001
Intermediate-risk, n (%)	140(19.9)	22(12.5)	37(21.0)	62(33.7)	19(11.3)	<0.001
High-risk, n (%)	221(31.4)	5(2.8)	10(5.7)	59(32.1)	147(87.5)	<0.001

WC, waist circumference; TG, triglycerides; TC, total cholesterol; HDL-c, high-density lipoprotein cholesterol; LDL-c, low-density lipoprotein cholesterol; SBP, Systolic blood pressure; DBP, Diastolic blood pressure; PrFT, perirenal fat thickness.

### Correlation of PrFT with Framingham and China-PAR equation risk scores

The univariate correlation analysis demonstrated a positive correlation between PrFT and Framingham equation risk scores, both in men (*r*=0.353, *P*<0.001) and women (*r*=0.408, *P*<0.001). Similarly, a positive association was observed between PrFT and China-PAR equation risk scores in both men (*r*=0.385, *P*<0.001) and women (*r*=0.375, *P*<0.001). Further investigation into these correlations was conducted through multiple linear regression analyses. As outlined in **Table 2**, positive correlations between PrFT and these risk scores persisted even after adjusting for Model 1 and Model 2 in both genders (*P*<0.001). Additionally, even after further adjustment for VFA and usage of GLP-1 RAs or SGL-T2is in Model 3, PrFT remained significantly correlated with Framingham equation risk scores in men (*β*=0.098, *P*=0.036) and women (*β*=0.099, *P*=0.032). Similarly, the correlation between PrFT and China-PAR equation risk scores persisted in men (*β*=0.106, *P*=0.009) and women (*β*=0.108, *P*=0.007).

**Table 2:** Multivariate linear regression analysis of the association between PrFT and the Framingham and China-PAR equation risk score.

Variable	Men (n=364)		Women (n=340)	
	*β*	*P*	*β*	*P*
Framingham risk score				
Model 1	0.314	<0.001	0.375	<0.001
Model 2	0.150	<0.001	0.171	<0.001
Model 3	0.098	0.036	0.099	0.032
China-PAR equation risk score				
Model 1	0.355	<0.001	0.393	<0.001
Model 2	0.159	<0.001	0.210	<0.001
Model 3	0.106	0.009	0.108	0.007

Model 1: adjustment for the diabetic duration, glycated hemoglobin A1c, homeostasis model assessment of insulin resistance, triglycerides, and low-density lipoprotein cholesterol.

Model 2: additional adjustment for age, waist circumference, total cholesterol, high-density lipoprotein cholesterol, systolic blood pressure, diastolic blood pressure, family ASCVD history, smoking, and urbanization.

Model 3: further adjustment for the visceral fat area and usage of glucagon-like peptide 1 receptor agonists or sodium-glucose co-transporters type 2 inhibitors.

PrFT, perirenal fat thickness.

### Correlation of PrFT with high estimated CVD and ASCVD risk

**Table 3** presents the results of the correlation of PrFT with high estimated 10-year CVD and ASCVD risk, analyzed by binomial logistic regression analysis. The results indicated that PrFT was independently associated with high estimated CVD and ASCVD risk after adjustment for Model 1 and Model 2 whether in men or women. Notably, these correlations remained significant after further adjustment for VFA and usage of GLP-1 RAs or SGL-T2is in Model 3, The ORs for a high estimated CVD risk were 1.14 (95% CI: 1.04-1.25, *P*=0.016) in men and 1.20 (95% CI: 1.11-1.31, *P*<0.001) in women, respectively. Concurrently, the ORs for a high estimated 10-year ASCVD risk were 1.22 (95% CI: 1.08-1.41, *P*=0.009) in men and 1.32 (95% CI: 1.12-1.60, *P*<0.001) in women, respectively.

**Table 3:** Binomial Logistic Regression Analysis adjusted ORs (95% CIs) for the associations between PrFT and high estimated 10-year risk of CVD and ASCVD.

Models	Men		Women	
	OR (95%CI)	*P*	OR (95%CI)	*P*
High estimated 10-year CVD risk				
Model 1	1.40(1.31-1.49)	<0.001	1.32(1.24-1.42)	<0.001
Model 2	1.34(1.24-1.44)	<0.001	1.26(1.17-1.36)	<0.001
Model 3	1.14(1.04-1.25)	0.016	1.20(1.11-1.31)	<0.001
High estimated 10-year ASCVD risk				
Model 1	1.51(1.38-1.64)	<0.001	1.56(1.39-1.74)	<0.001
Model 2	1.44(1.31-1.58)	<0.001	1.51(1.35-1.70)	<0.001
Model 3	1.22(1.08-1.41)	0.009	1.34(1.12-1.60)	<0.001

Model 1: adjustment for the diabetic duration, glycated hemoglobin A1c, homeostasis model assessment of insulin resistance, triglycerides, and low-density lipoprotein cholesterol.

Model 2: additional adjustment for age, waist circumference, total cholesterol, high-density lipoprotein cholesterol, systolic blood pressure, diastolic blood pressure, family ASCVD history, smoking, and urbanization.

Model 3: further adjustment for the visceral fat area and usage of glucagon-like peptide 1 receptor agonists or sodium-glucose co-transporters type 2 inhibitors.

PrFT, perirenal fat thickness.

### Non-linear relationship of PrFT with high estimated CVD and ASCVD risk

**Figure 2:**
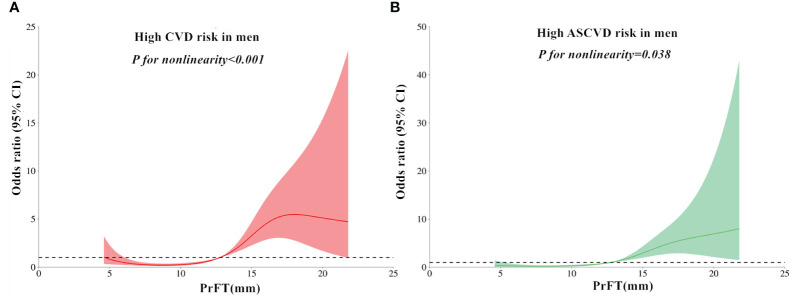
Restricted cubic spines analysis of the association between PrFT and high estimated 10-year risk of CVD **(A)** and ASCVD **(B)** after adjustment for Model 3 in men. PrFT, Perirenal fat thickness; CVD, Cardiovascular disease; ASCVD, Atherosclerotic cardiovascular disease.

**Figure 3:**
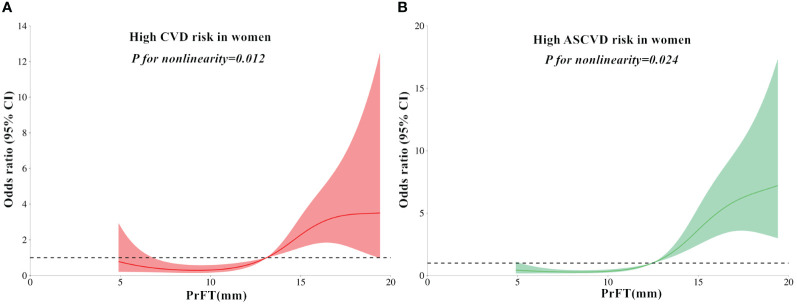
Restricted cubic spines analysis of the association between PrFT and high estimated 10-year risk of CVD **(A)** and ASCVD **(B)** after adjustment for Model 3 in women. PrFT, Perirenal fat thickness; CVD, Cardiovascular disease; ASCVD, Atherosclerotic cardiovascular disease.

The RCS analyses were conducted to elucidate further the relationship between PrFT and the high estimated risks of CVD and ASCVD. Following adjustment for Model 3, the results revealed a non-linear correlation of PrFT with high estimated CVD (*P* for nonlinearity < 0.001) and ASCVD risk (*P* for nonlinearity = 0.038) in men (**Figure 2**). Similarly, the non-linear correlation of PrFT with high estimated CVD (*P* for nonlinearity = 0.012) and ASCVD risk (*P* for nonlinearity=0.024) in women persisted (**Figure 3**).

## Discussion

Recent evidence in CVD management has underscored the potential significance of PAT accumulation as a pivotal factor in CVD pathogenesis. This highlights the possibility of PAT as a viable treatment target for CVD. This study evaluated the association between increased PrFT and estimated 10-year CVD and ASCVD risk from a clinical perspective. The findings revealed increasing trends in the Framingham and China-PAR equation risk scores with higher PrFT quartiles. Additionally, PrFT was independently correlated with Framingham and China-PAR equation risk scores. Furthermore, PrFT contributes as an independent variable to the high estimated risk of CVD and ASCVD.

Obesity and T2DM stand as significant contributors to CVD, a condition rapidly escalating on a global scale. Given the shared risk factors between T2DM and obesity, including hypertension, dyslipidemia, and insulin resistance, their co-occurrence significantly amplifies CVD risk. Particularly, abdominal obesity plays a pivotal role in the pathogenesis of CVD, with VAT accumulation showing a stronger correlation with CVD risk compared to subcutaneous fat (19, 20). Recent attention has turned towards PAT in elucidating its role in CVD. Situated within the retroperitoneal space surrounding the kidneys, PAT possesses distinct anatomical and functional characteristics distinguishing it from typical visceral fat depots. Unlike conventional visceral fat, PAT shares similarities with internal organs, boasting a complete blood supply, lymphatic drainage, and innervation (21). These unique features equip PAT with a dynamic capacity in energy metabolism and adipokine modulation, rendering it a potential modulator of the cardiovascular system through autonomic nervous system regulation. Moreover, PAT serves as a robust source of adipokines such as leptin, adiponectin, apelin, and nestin, which exert systemic effects on cardiovascular, immune, and metabolic regulation (22). Additionally, local immune cells within PAT synthesize cytokines like tumor necrosis factor-alpha and interleukin-6, which are implicated in CVD pathogenesis (23). Given these intricate regulatory mechanisms, the excessive accumulation of PAT is hypothesized to heighten CVD risk.

PrFT has emerged as a validated index adept at accurately representing PAT mass, extensively utilized for exploring the correlation between elevated PAT and CVD risk factors. Sahin et al. observed a significant positive correlation between PrFT and both SBP and DBP in patients diagnosed with polycystic ovary syndrome (24). Additionally, Campobasso et al. found a positive association between PrFT and mean 24-hour SBP levels in overweight and obese subjects (25). Meanwhile, PrFT was also correlated with other cardio-metabolic risk factors such as WC, TG, HDL-c, and HOMA-IR (26, 27). Recent studies have observed close associations between increased PrFT and increased intima-media thickness (16, 28, 29), cardiac hypertrophy (30), and systemic calcified atherosclerosis (31), which were considered predictors of future CVD events. Consistent with previous studies, this study revealed significant differences in cardiometabolic risk across the PrFT quartiles, encompassing WC, TG, TC, HDL-c, and HOMA-IR. These findings underscore a close correlation between increased PAT and CVD risk factors.

Risk assessment constitutes a cornerstone in the prevention of CVD or ASCVD. Precise evaluation of individual risk is pivotal in guiding and facilitating preventive measures against CVD or ASCVD. Framingham risk scores were derived from the extensive Framingham Heart Study data, designed to estimate 10-year CVD risk in individuals aged 30 to 74. China-PAR equation risk scores, developed from data specific to the prediction of ASCVD risk in China, have undergone validation and application in prior research (32, 33). Xu et al. revealed that VFA contributed to the Framingham 10-year general cardiovascular disease risk after statistical correction for other multiple factors affecting CVD risk in 202 participants with T2DM (34). This study further adjusted the VFA in addition to other cardiometabolic factors affecting CVD risk in previous studies. The findings revealed that increased PrFT was independently and positively correlated with Framingham and China-PAR equation risk scores. Furthermore, PrFT also contributes as an independent variable to the high estimated CVD and ASCVD risk. While prior studies showed sex-specific associations between BMI and CVD risk (35), this study revealed a positive correlation of PrFT with estimated CVD or ASCVD risk regardless of gender. These findings indicate that increased PAT accumulation was significantly associated with high CVD or ASCVD risk. Maria et al. observed reduced requirements for antihypertensive medications and systolic blood pressure (SBP) levels in hypertensive obese subjects following sleeve-gastrectomy surgery, associated with decreased PAT (14). GLP-1 RAs and SGL-T2i exhibit recognized CVD prevention properties, with clinical studies demonstrating reduced PAT after these medications (36, 37). It is plausible that the cardiovascular protective effect of GLP-1 RAs, SGL-T2i, or sleeve-gastrectomy surgery partially involves reducing PAT. Furthermore, renal sinus fat (RSF) has garnered clinical interest. Catharine et al. discovered a positive correlation between RSF and DBP while noting an inverse relationship with insulin sensitivity (38). Similarly, Moritz et al. demonstrated that RSF expansion is prevalent in individuals with obesity and/or hypertension, but can be reduced through bariatric surgery, subsequently associated with the remission of hypertension (39). These insights strongly suggest that RSF might contribute significantly to the onset of hypertension and CVD. Therefore, heightened attention to perirenal and intrarenal fat deposition is warranted due to its potential role in CVD.

## Strength and limitation

To the best of our knowledge, this study is the first to investigate the association between increased PrFT and estimated 10-year CVD and ASCVD risk. However, it is important to acknowledge certain limitations in this study. The study population was limited to individuals with T2DM in southern China, which may restrict the generalizability of the findings to other ethnicities or populations residing in northern China. Additionally, the cross-sectional study design, based on prospectively gathered data from a single center, restricts the capacity to establish causality between heightened PrFT and elevated estimated CVD and ASCVD risk. Future research involving diverse populations and longitudinal designs will offer valuable insights into this association. Furthermore, as we assessed PrFT using CT scans, it is important to note that radiation exposure may limit its clinical utility, especially in pregnant women and children.

## Conclusion

This study demonstrated a positive correlation between increased PrFT and Framingham and China-PAR equation risk scores. Additionally, PrFT independently contributed as a variable to the high estimated 10-year risk of CVD and ASCVD in individuals with T2DM. These findings suggest that increased PAT may serve as a risk factor for CVD. Future investigations should concentrate on identifying strategies to reduce CVD risk by targeting PAT as a potential therapeutic target.

## Data availability statement

The original contributions presented in the study are included in the article/supplementary material. Further inquiries can be directed to the corresponding author.

## Ethics statement

The studies involving humans were approved by the Ethical Committee of Longyan First Affiliated Hospital of Fujian Medical University (IC-2020-069). The studies were conducted in accordance with the local legislation and institutional requirements. The participants provided their written informed consent to participate in this study.

## Author contributions

WW: Data curation, Formal analysis, Investigation, Methodology, Project administration, Software, Writing – original draft. FL: Data curation, Investigation, Methodology, Writing – original draft. MT: Data curation, Investigation, Methodology, Writing – original draft. XG: Conceptualization, Data curation, Formal analysis, Investigation, Methodology, Writing – review & editing.
